# Assessment of Molecular Residual Disease Using Circulating Tumor DNA to Identify Multiple Myeloma Patients at High Risk of Relapse

**DOI:** 10.3389/fonc.2022.786451

**Published:** 2022-02-02

**Authors:** Binod Dhakal, Shruti Sharma, Mustafa Balcioglu, Svetlana Shchegrova, Meenakshi Malhotra, Bernhard Zimmermann, Paul R. Billings, Alexandra Harrington, Himanshu Sethi, Alexey Aleshin, Parameswaran N. Hari

**Affiliations:** ^1^ Division of Blood and Marrow Transplant (BMT) & Cellular Therapy, Medical College of Wisconsin, Milwaukee, WI, United States; ^2^ Oncology, Natera, Inc., Austin, TX, United States; ^3^ Department of Pathology, Medical College of Wisconsin, Milwaukee, WI, United States

**Keywords:** circulating tumor DNA (ctDNA), tumor-informed, minimal/molecular residual disease, multiple myeloma (MM), multiparameter flow cytometry (MFC)

## Abstract

**Background:**

Despite treatment with high-dose chemotherapy followed by autologous stem cell transplantation (AHCT), patients with multiple myeloma (MM) invariably relapse. Molecular residual disease (MRD)-negativity post-AHCT has emerged as an important prognostic marker predicting the duration of remission. Current techniques for MRD assessment involve bone marrow (BM) aspirate sampling, which is invasive, subject to sample variability and is limited by spatial heterogeneity. We compared the performance of a non-invasive, circulating tumor DNA (ctDNA)-based MRD assay with multiparameter flow cytometry (MFC) of marrow aspirate to predict relapse in AHCT recipients with MM.

**Methods:**

MRD assessment using ctDNA was retrospectively analyzed on 80 plasma samples collected at different time points from 28 patients, post-AHCT. MFC was used to assess MRD from BM biopsy. Individual archived BM aspirate slides or formalin-fixed paraffin-embedded slides from the time of MM diagnosis and matched blood were used to assess MRD at 3 months, post-AHCT, using a personalized, tumor-informed ctDNA assay.

**Results:**

ctDNA was detectable in 70.8% (17/24) of pre-AHCT patients and 53.6% (15/28) of post-AHCT patients (3-month time point). Of the 15 post-AHCT ctDNA-positive patients, 14 relapsed on follow-up. The median PFS for ctDNA-positive patients was 31 months, and that for ctDNA-negative patients was 84 months (HR: 5.6; 95%CI: 1.8-17;p=0.0003). No significant difference in PFS was observed in patients stratified by MFC-based MRD status (HR 1.2; 95%CI: 0.3-3.4;p=0.73). The positive predictive value for ctDNA was also significantly higher than MFC (93.3% vs. 68.4%).

**Conclusions:**

This study demonstrates tumor-informed ctDNA analysis is strongly predictive of MM relapse.

## Introduction

Multiple myeloma (MM) is the second most common hematological cancer in the US, and despite the dramatic changes in treatment, remains incurable (1) The advancements in treatment options have led to an increased proportion of patients achieving better outcomes (2). Minimal/molecular residual disease (MRD) assessment using next-generation sequencing (NGS) and next-generation flow (NGF) have the ability to detect MRD at 10^-5^ and 10^-6^ levels. The prognostic significance of MRD, regardless of the treatment and methodology, has been well established for both newly diagnosed MM as well as relapsed and/or refractory MM (3). Furthermore, undetectable MRD has shown to overcome the poor prognosis of high-risk cytogenetics in MM, as these patients have similar survival when compared with MRD-negative standard risk patients (4). These observations underscore the prognostic significance of MRD and its need to be accepted as a clinical end-point in MM.

However, bone marrow (BM)-based MRD assessment has several limitations due to patchy quality and hemodilution of BM samples, false negative results due to extramedullary disease and invasive nature of the procedure (5). Furthermore, the feasibility of monitoring the genetic characteristics due to the spatial and temporal heterogeneity resulting from tumor evolution and progression is challenging (6).

Circulating tumor DNA (ctDNA) has emerged as a promising noninvasive biomarker for longitudinal assessment of solid tumors (7, 8). Detection and quantification of ctDNA provides an accurate assessment of tumor burden beyond other available serological markers (8, 9). Here we evaluated the prognostic role of ctDNA-based MRD detection using a personalized multiplex amplicon-based NGS platform on archival samples in MM patients after autologous hematopoietic cell transplantation (AHCT).

## Materials and Methods

### Patients and Samples

A total of 80 plasma samples from 28 patients with MM were analyzed retrospectively at Medical College of Wisconsin. This sample set is representative of the Multiple Myeloma patients. High risk FISH patients were defined by t (4;14), t(14;16), t (14;20), 17p deletion) and 1q gain/amplification, and 1p deletion. All patients had upfront AHCT, following initial induction on the BMT CTN 0702 STaMINA study. All patients were on lenalidomide maintenance, post-AHCT. Plasma samples were available for 3 time points: pre-AHCT, at 3 months and ~1 year post AHCT. The primary objective of the study was to correlate ctDNA status at 3 months post-AHCT with progression-free survival (PFS). The secondary objective was to compare the performance of ctDNA-based MRD assessment multiparameter flow cytometry (MFC) analysis. The study was performed in accordance with the Declaration of Helsinki and all participants provided written informed consent.

### MRD Assessment Using ctDNA (mPCR-NGS Methodology) and Multiparameter Flow Cytometry

MRD assessment at 3 months post-AHCT was performed by ctDNA analysis using a personalized, tumor-informed (Signatera™, bespoke mPCR-NGS assay). Briefly, whole exome sequencing (WES) was performed on Formalin-Fixed Paraffin Embedded (FFPE) tumor blocks or individual bone marrow aspirate (BMA) slides (available from the time of MM diagnosis) and matched germline DNA obtained from blood samples. On analyzing the WES sequencing results, primers were designed for up to 16 patient-specific somatic clonal SNVs for mPCR testing, which were subsequently used to identify and track ctDNA in the patient’s plasma. ctDNA can be detected with both a high sensitivity and high specificity, reliably detecting variants down to 0.01% VAF (7). Cell-free DNA (cfDNA) was extracted from frozen plasma samples (2 mL) obtained at predetermined time points (pre-transplant: median 9.9 ng/mL, range: 1.2-315.5 ng/mL; post-transplant: median 8.8 ng/mL, range: 2.8–114.5 ng/mL). The presence of ctDNA was quantified in mean tumor molecules (MTM) per mL of plasma.

MFC was used to assess MRD from BM biopsy and was assessed at 3 months post AHCT using institutional flow at 10^-4^ sensitivity (10). Briefly, 4- and 8-color flow cytometry was performed using a FACSCanto or FACSCalibur analyzer (BD Biosciences, San Jose, California) and fluorescent-labeled antibodies against CD19, CD20, CD38, CD45, CD56, CD117, CD138, CD200, and/or CD319 (BD Biosciences). Surface and intracytoplasmic light chain expression were determined using kappa and lambda antibodies (BD Biosciences); abnormal light chain ratios were defined as <0.5 and >4. MRD analysis consisted of 200,000 to 500,000 acquired events/tube, analyzed by a hematopathologist using Paint-A-Gate software (BD Biosciences), with a sensitivity of 0.01%.

### Statistical Analysis

Survival analyses were performed using the Kaplan-Meier Estimator and the Cox method. These analyses were carried out in R version 3.6.1 using packages survminer, survival, and coxphf (https://cran.r-project.org). Local MRD evaluation by MFC 10^-4^ level was compared with the ctDNA assay. The prognostic value of ctDNA was evaluated by correlating MRD status with clinical outcomes (PFS) using Cox regression. PFS was defined as the time from AHCT until progression or death. High risk cytogenetics was defined as having one of the following: t (4;14), t (14;16), t (14;20), 17p deletion, 1q gain and 1p deletion. A multivariable Cox proportional hazards model was employed to assess the most significant prognostic factor associated with PFS. For multivariate analysis, the age, cytogenetics and MRD status by MFC were included as covariates along with ctDNA status. All *p* values were based on 2-sided testing, and differences were considered significant at *p ≤* 0.05. The ctDNA statistical analysis plan was developed before unblinding of the clinical data and followed for the analysis. The data assessors were blinded to patient outcome and sample order. Neither treating clinicians nor patients were informed about the ctDNA results.

## Results

**Figure 1:**
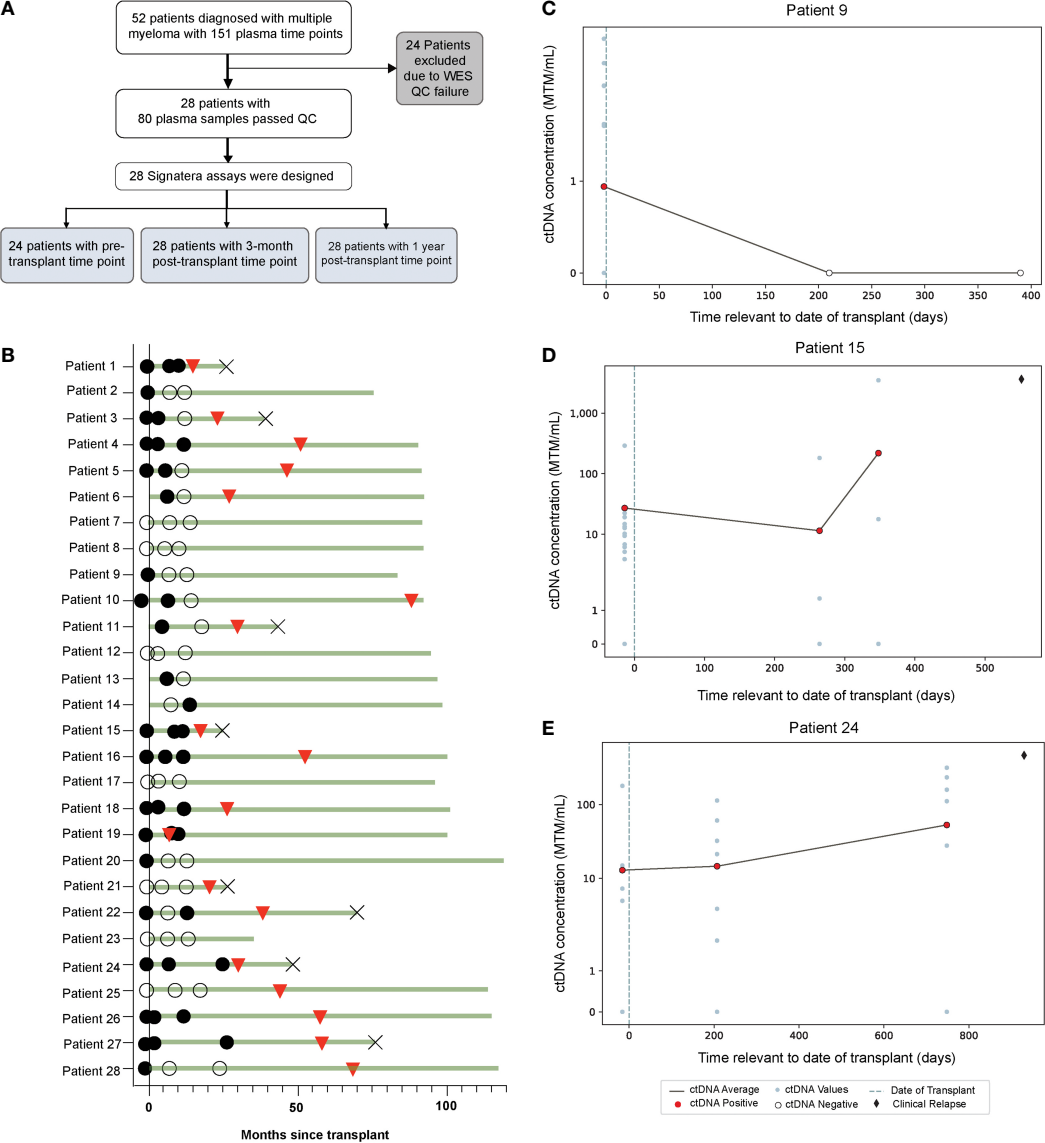
**(A)** Study design and **(B)** Overview plot across different time points, illustrating association of ctDNA (filled circles: ctDNA positive, clear circles: ctDNA negative) with clinical response (red triangles: clinical relapse, cross: death). **(C–E)** Target plots of ctDNA status for individual patients.

A total of 52 patients with 151 available plasma time points were eligible for the study. Of these, 24 patients were excluded due to tissue WES quality check (QC) failure due to insufficient quantity/quality of DNA from archival (~10-15 years in age) BMA slide or FFPE tissue slide samples. The remaining 28 patients with 80 plasma samples passed WES QC parameters and were included in the study (**Figure 1A**). As shown, only 24 patients had plasma samples available before the transplant. Baseline patient characteristics are shown in **Table 1**. The median age of the patients was 67 years (range, 41-70) and 57% were males. High risk cytogenetics was present in 14% of the patients. About 50% of the patients achieved complete response after AHCT.

**Table 1:** Baseline patient characteristics.

Characteristics	N=28 (%)
**Age, median (range), years**	67 (41-70)
**Gender**	
Male	16 (57.1)
**Race**	
White	24 (86)
AA	3 (11)
Others	1 (3)
**Isotype**	
IgG kappa/lambda	20 (71)
IgA kappa/lambda	3 (11)
Light chain	5 (18)
**ISS stage^1^**	
I	15 (54)
II	11 (39)
III	1 (3.5)
Unknown	1 (3.5)
**High risk cytogenetics^2^**	4 (14)
**Induction^3^**	
VRD	13 (46)
CyBorD	11 (39)
Others	4 (15)
**Maintenance**	
Lenalidomide	28 (100)
**Time from diagnosis to transplant**	
**Median, months (range)**	6 (4-8)
**Post-Transplant IMWG response**	
Complete/near complete response	14 (50)
Very good partial response	5 (18)
Partial response	9 (32)

1. International staging system.

2. t (4;14), t (14;16), t (14;20), 17p deletion, 1q amplification and 1p deletion.

3. VRD: bortezomib, lenalidomide and dexamethasone; CyBorD: Cyclophosphamide, bortezomib and dexamethasone.

IMWG, International myeloma working group.

### MRD Assessment Using ctDNA (mPCR-NGS Methodology) and MFC

In this cohort, a total 64.2% (18/28) of patients experienced relapse (**Figure 1B**). ctDNA was detectable in 70.8% (17/24) of pre-AHCT, 53.6% (15/28) of post-AHCT, and 39.2% (11/28) of patients at the 1-year follow-up time point (**Figure 1B**). ctDNA target plots for individual patients are represented in (**Figures 1C–E**). We chose to highlight longitudinal ctDNA results from patients 9, 15, and 24 because they are particularly illustrative of the clinical utility of personalized and tumor-informed ctDNA testing. Patient 9 tested ctDNA-positive prior to AHCT, and tested ctDNA-negative twice thereafter, up to ~400 days post-transplant. The patient did not experience clinical relapse. Patients 15 and 24 tested ctDNA-positive prior to AHCT and continued to stay positive, suggesting the presence of MRD. In both cases, ctDNA levels further increased at the third time point. Approximately 100 days (patient 24) and 200 days (patient 15) after ctDNA levels increased, both patients went on to relapse, confirmed by imaging. These cases demonstrate that ctDNA-positivity at the MRD time point is not only prognostic, but can predict relapse far ahead of clinical progression. MRD-positivity by MFC is shown in **Supplementary Figure 1**.

**Figure 2:**
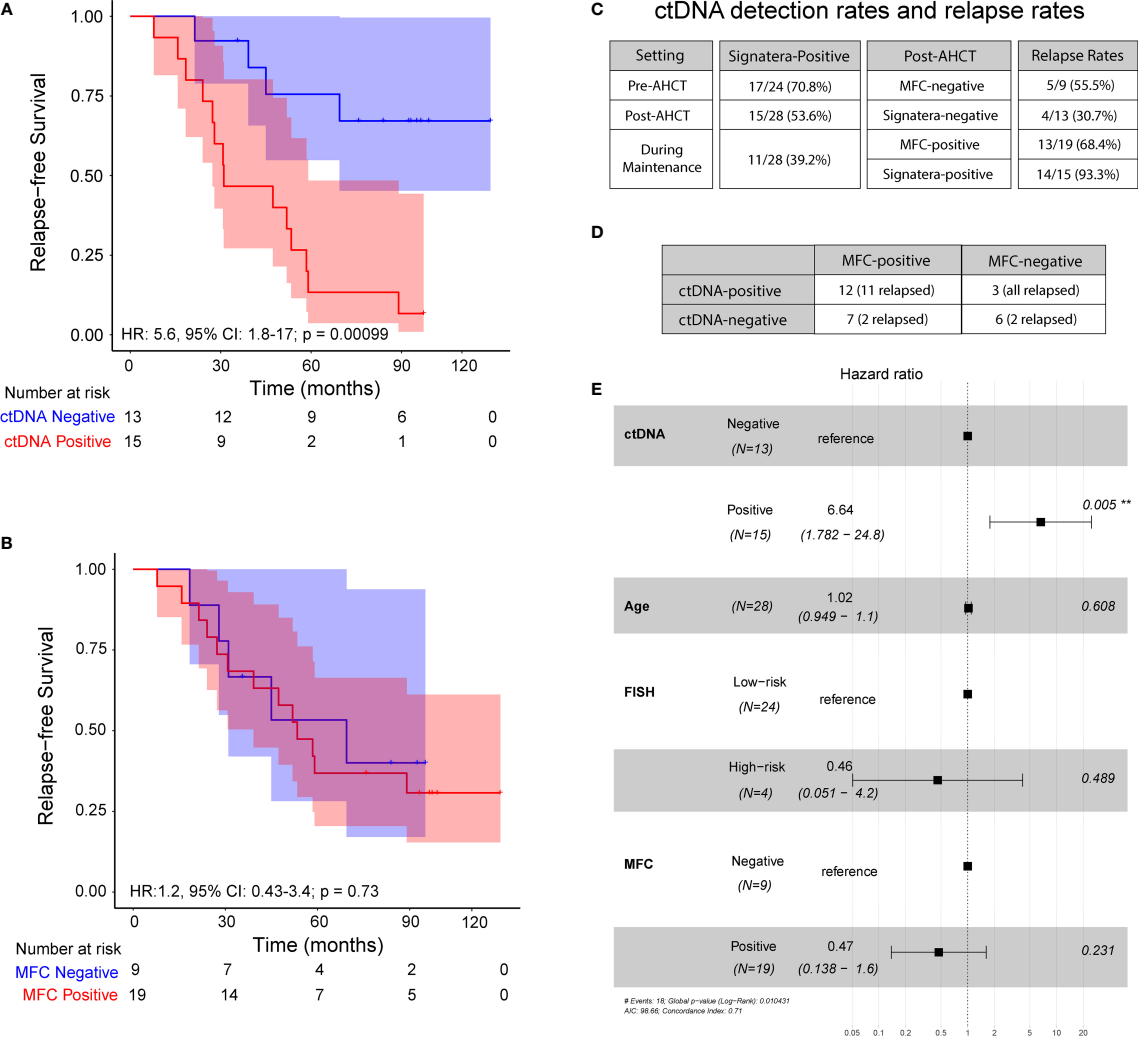
Progression-free survival at 3 – month post-transplant: **(A)** ctDNA analysis in peripheral blood and **(B)** Multiparameter flow cytometry at 10^-4^ sensitivity. **(C)** ctDNA detection rates and relapse rates across the cohort. **(D)** Analysis comparing MFC status to ctDNA status, both measured 3 months post-transplant. **(E)** Multivariate analysis of prognostic factors and their association with progression-free survival, as indicated by hazard ratio, analyzed across the cohort.

Overall, at 3 months post AHCT, 15 (54%) patients were ctDNA positive and remaining 13 (46%) were ctDNA negative whereas 19 (68%) patients were MRD-positive by MFC and 9 (32%) were MRD-negative by MFC. At a median follow-up of 52.65 months, ctDNA-negative patients at 3 months post-AHCT had significantly longer PFS than ctDNA-positive patients [median 84 months (range: 21.3-119.5) vs. 31 months (range: 7.8- 97.3); HR 5.6; 95% CI: 1.8-17; p=0.0003] (**Figure 2A**). On the other hand, no significant difference was observed in PFS in patients stratified by MFC-based MRD status [median PFS in MRD-negative patients (44.96 months, range: 18.4-95.1) vs. median PFS in MRD-positive patients (53.4 months, range: 7.83-119.5); HR 1.2, 95% CI: 0.3-3.4; p=0.73] (**Figure 2B**).

Of the 15 ctDNA-positive patients at the 3-month post-AHCT, 14 (93.3%) patients progressed and of 19 patients who were MRD-positive by MFC at the same time point, only 13 (68.4%) patients showed evidence of disease progression. Similarly, of 13 patients who were ctDNA-negative, 4 (30.7%) patients relapsed at the last follow-up, while 5 of the 9 (55.5%) patients who were negative by MFC at 3 months had disease progression. At this time point, ctDNA analysis revealed a positive predictive value (PPV) of 93.3% and negative predictive value (NPV) of 69.3% (**Figure 2C**). The confidence limits for relapse rate in MFC-negative cohort using a simple binomial test comes out to be 21.2% < 55.5% < 86.3%. While the measured relapse rate of 30.7% for the ctDNA-negative cohort, does fall within the confidence interval, it is close to the lower bound. This indicates that the negative predictive value of the ctDNA method is at least noninferior to MFC, with a good margin. On the other hand, the confidence limits for the relapse rate in the MFC-positive cohort are 43.5% < 68.4% < 87.4%. The measured relapse rate in the ctDNA-positive cohort is 93.3% and lies well outside these confidence limits.

On comparing, ctDNA and MFC data, we observed a concordance of 64.2% (18/28) and a discordance of 35.8% (10/28) (**Figure 2D**). Among the discordant cases, 100% of the ctDNA-positive patients that were negative by MFC (n=3) relapsed. On the other hand, of the 7 patients that were positive by MFC but negative for ctDNA, only 2 patients relapsed (28.6%). Collectively, these results suggest greater sensitivity of personalized ctDNA assay over MFC in accurately predicting relapse.

In multivariate analysis, ctDNA status at 3 months was found to be an independent prognostic factor (hazard ratio, HR 8.86, 95% CI: 1.83-43; p<0.007 (**Figure 2E**) for PFS. MRD status by MFC had no impact on outcomes as shown (HR 0.47; 95% CI: 0.13-1.6; p= 0.23).

## Discussion

This study of uniformly treated upfront AHCT patients with newly diagnosed MM aimed to examine the feasibility and predictive ability of a ctDNA-based MRD assay in the post-transplant setting using archived blood and marrow samples. Our study showed that ctDNA-positivity at the 3-month time point post AHCT was predictive of progression and superior to MFC at 10^-4^ sensitivity.

Other reports have shown the prognostic value of MRD testing at a single time point (11, 12). Medina et al. compared the prognostic value of MRD testing 3 months post-transplant, evaluated by NGS and NGF. In both assays, MRD-negative patients showed a significantly better 3-year PFS rate than the positive patients (*p* < 0.001), either basing on NGS (88.7% vs. 56.6%) or NGF results (91.4% vs. 50%), respectively, *p* < 0.001 (12).

MRD assessment using peripheral blood (PB) has been investigated in MM using circulating plasma cells (CPCs), digital droplet PCR-based ctDNA assessment and mass spectrometry (MS) analysis (13–15). Findings from these studies suggest convenience of using PB for MRD assessment, however, these methodologies are not as sensitive as marrow based NGS or NGF methodologies (16, 17). In the first comparative study of MRD by NGS on immunoglobulin (Ig) gene rearrangements between ctDNA and bone marrow, MRD burden in ctDNA was not correlated to bone marrow suggesting a limited quantitative significance of ctDNA (18). Another study using deep sequencing demonstrated a clear correlation between myeloma clone levels in paired bone marrow and peripheral blood samples; however, the assay demonstrated superior sensitivity in the bone marrow compartment (19). Both these studies that have used an NGS-based approach for identifying and quantifying Ig gene rearrangements underscore the need for additional analysis to understand the utility of blood in monitoring MM. This could potentially be achieved by using an assay that employs additional molecular targets.

In this regard, our study employs a personalized, tumor-informed ctDNA test that detects and quantifies up to 16 tumor-derived, clonal, somatic single nucleotide variants for the purpose of MRD detection. Having the patient’s archival FFPE samples and BMA slides, from which DNA can be extracted, allows WES to be performed. WES identifies the somatic variants present in the tumor versus matched normal tissue, from which clonal variants specific to the individual patient’s tumor are selected. Most clonal variants are passenger mutations, i.e. mutations that have no effect on the fitness of a clone but are associated with clonal expansion, and are not susceptible to treatment-induced attrition. Thus, clonal variants are truly representative of the overall tumor burden over time, and can be tracked to measure disease progression/treatment response with high confidence. By identifying and tracking clonal variants, which are expected to be present in every cancer cell from the patient, the tumor-informed approach, as used in this study (bespoke mPCR-NGS ctDNA assay) ensures that residual disease can be detected with both a high sensitivity and high specificity. The tumor-informed method also significantly reduces the false-positive rates by filtering out clonal hematopoiesis of indeterminate potential (CHIP) and germline-derived variants from analysis. Compared to other fixed panel-based ctDNA technologies that are limited to 0.1%-1% plasma-level variant allele frequency (VAF) detection, the ctDNA assay used in this study has previously demonstrated >95% sensitivity at (VAF) of 0.01% (7). In this study, the ctDNA assay has demonstrated a PPV and NPV of 93.3% and 69.3%, respectively, which is encouraging when compared to prior studies. However, it is important to realize that the MFC-based MRD assay was at a sensitivity level of 10^-4^. Although, it is a standard BM-based MRD test that is used in the clinical setting, it is not as sensitive as an NGS and next generation flow (NGF)-based assay.

The major limitation of our study stems from the fact that the initial sample collection was not intended for this study and the archival marrow samples did not yield sufficient DNA quantity or quality to enable the design of the tumor-informed ctDNA assay for 46% of the patients. Samples were more than 10 years old, which likely contributed to the high extraction failure rates. Sufficient DNA quantity/quality is needed in order to successfully perform WES, the results of which are a requirement for the selection of the 16 SNVs for personalized and tumor-informed ctDNA testing. In all cases where sufficient DNA quantity/quality was extracted for archival FFPE or BMA samples (n=28), we were able to successfully design patient-specific primers and perform ctDNA testing for 100% of the patients. In real world practice, we anticipate sample extraction failure rates to be less than 3%, which was observed in a prospective observational study (GALAXY study - UMIN000039205 in CIRCULATE-Japan trial) being conducted in stage II-IV colorectal cancer patients (20).

Dedicated marrow samples and germline DNA collected at initial diagnosis may enhance the feasibility of the test and its clinical utility. We acknowledge that in this study, we were limited by a relatively small sample size and the less sensitive (10^-4^) MFC based MRD assay, which was the only MRD assay available during that period. We note that the lack of significance in the PFS data for patients stratified by MFC-based MRD could be because of the nature of a less sensitive assay and that it would have been of value to compare the sensitivity of our multi-target ctDNA assay, which employs an amplicon-based sequencing (average depth per amplicon of >100,000) with a commercially available NGS assay that has a comparable performance.

On a positive note, with the use of a personalized mPCR-NGS methodology, we were able to design and track clonal variants in archival samples. In future studies, we plan to compare the performance of personalized mPCR-NGS ctDNA testing to that of BM-derived NGS and NGF technologies, for early detection of relapse. We believe that the personalized mPCR-NGS ctDNA testing that identifies clonal variants truly represents the overall tumor burden, which can be tracked over time and will likely overcome the limitations associated with localized BM-based MRD assays, which may not capture the overall disease burden. Overall, this study indicates a strong potential to improve the clinical management of MM with the introduction of personalized ctDNA analysis. This can further be strengthened by replicating the results in additional cohorts.

## Data Availability Statement

The original contributions presented in the study are included in the article/**Supplementary Material**. Further inquiries can be directed to the corresponding author.

## Ethics Statement

This study was conducted in accordance with the World Medical Association's Declaration of Helsinki policy. All patients/participants provided written informed consent to participate in the study.

## Author Contributions

Conceptualization – BD, PH, AA, and BZ. Data acquisition/curation – HS, MB, SvS, BD, and AH. Data analysis/interpretation – SvS, ShS, and AA. Writing - original draft – BD, ShS, and MM. Writing - reviewing and editing –BD, ShS, MB, SvS, MM, BZ, PB, AH, HS, AA, and PH. All authors contributed to the article and approved the submitted version.

## Conflict of Interest

BD has received honoraria from Celegene/BMS, Karyopharm, GSK and Sanofi; serves on the ad board for Takeda, Amgen, Janssen, GSK, Natera, and Arcellx; has received research support from Takeda, Amgen, Janssen, GSK, Sanofi. Carsgen. Arcellx and Cartesian. PH has received honoraria from Amgen, BMS, Janssen, Sanofi and Takeda; serves on the ad board for Millenium; serves as a consultant for Amgen, BMS, GSK, Karyopharm and Takeda; has received research support from Amgen, BMS, Celgene, GSK, Millenium, Sanofi and Takeda. ShS, MB, SvS, MM, BZ, PB, HS, and AA are employees of Natera, Inc. with stock/options to own stock in the company.

The remaining author declares that the research was conducted in the absence of any commercial or financial relationships that could be construed as a potential conflict of interest.

## Publisher’s Note

All claims expressed in this article are solely those of the authors and do not necessarily represent those of their affiliated organizations, or those of the publisher, the editors and the reviewers. Any product that may be evaluated in this article, or claim that may be made by its manufacturer, is not guaranteed or endorsed by the publisher.

## References

[1] Van De Donk N Pawlyn C Yong KL. Multiple Myeloma. Lancet (2021) 397:410–27. doi: 10.1016/S0140-6736(21)00135-5 33516340

[2] Anderson KC Alsina M Atanackovic D Biermann JS Chandler JC Costello C. Multiple Myeloma, Version 2.2016. J Natl Compr Cancer Netw (2015) 13:1398–435. doi: 10.6004/jnccn.2015.0167 PMC4891187 26553768

[3] Munshi NC Avet-Loiseau H Anderson KC Neri P Paiva B Samur M. A Large Meta-Analysis Establishes the Role of MRD Negativity in Long-Term Survival Outcomes in Patients With Multiple Myeloma. Blood Adv (2020) 4:5988–99. doi: 10.1182/bloodadvances.2020002827 PMC7724898 33284948

[4] Goicoechea I Puig N Cedena M-T Burgos L Cordón L Vidriales M-B. Deep MRD Profiling Defines Outcome and Unveils Different Modes of Treatment Resistance in Standard-and High-Risk Myeloma. Blood (2021) 137:49–60. doi: 10.1182/blood.2020006731 32693406

[5] Costa LJ Derman BA Bal S Sidana S Chhabra S Silbermann R. International Harmonization in Performing and Reporting Minimal Residual Disease Assessment in Multiple Myeloma Trials. Leukemia (2021) 35:18–30. doi: 10.1038/s41375-020-01012-4 32778736

[6] Rasche L Chavan S Stephens O Patel P Tytarenko R Ashby C. Spatial Genomic Heterogeneity in Multiple Myeloma Revealed by Multi-Region Sequencing. Nat Commun (2017) 8:1–11. doi: 10.1038/s41467-017-00296-y 28814763 PMC5559527

[7] Coombes RC Page K Salari R Hastings RK Armstrong A Ahmed S. Personalized Detection of Circulating Tumor DNA Antedates Breast Cancer Metastatic Recurrence. Clin Cancer Res (2019) 25:4255–63. doi: 10.1158/1078-0432.CCR-18-3663 30992300

[8] Cescon DW Bratman SV Chan SM Siu LL. Circulating Tumor DNA and Liquid Biopsy in Oncology. Nat Cancer (2020) 1:276–90. doi: 10.1038/s43018-020-0043-5 35122035

[9] Levin A Hari P Dhakal B. Novel Biomarkers in Multiple Myeloma. Trans Res (2018) 201:49–59. doi: 10.1016/j.trsl.2018.05.003 30301522

[10] Dhakal B D’souza A Martens M Kapke J Harrington AM Pasquini M. Allogeneic Hematopoietic Cell Transplantation in Multiple Myeloma: Impact of Disease Risk and Post Allograft Minimal Residual Disease on Survival. Clin Lymphoma Myeloma Leukemia (2016) 16:379–86. doi: 10.1016/j.clml.2016.03.001 27160644

[11] Landgren O Devlin S Boulad M Mailankody S. Role of MRD Status in Relation to Clinical Outcomes in Newly Diagnosed Multiple Myeloma Patients: A Meta-Analysis. Bone Marrow Transplant (2016) 51:1565–8. doi: 10.1038/bmt.2016.222 PMC5571752 27595280

[12] Medina A Puig N Flores-Montero J Jimenez C Sarasquete M-E Garcia-Alvarez M. Comparison of Next-Generation Sequencing (NGS) and Next-Generation Flow (NGF) for Minimal Residual Disease (MRD) Assessment in Multiple Myeloma. Blood Cancer J (2020) 10:1–10. doi: 10.1038/s41408-020-00377-0 33127891 PMC7603393

[13] Pugh TJ. Circulating Tumour DNA for Detecting Minimal Residual Disease in Multiple Myeloma. In: Seminars in Hematology. Elsevier. (2018) 55:38–40. 29759151 10.1053/j.seminhematol.2018.03.002

[14] Sanoja-Flores L Flores-Montero J Puig N Contreras-Sanfeliciano T Pontes R Corral-Mateos A. Blood Monitoring of Circulating Tumor Plasma Cells by Next Generation Flow in Multiple Myeloma After Therapy. Blood (2019) 134:2218–22. doi: 10.1182/blood.2019002610 PMC6966491 31697808

[15] Eveillard M Rustad E Roshal M Zhang Y Ciardiello A Korde N. Comparison of MALDI-TOF Mass Spectrometry Analysis of Peripheral Blood and Bone Marrow-Based Flow Cytometry for Tracking Measurable Residual Disease in Patients With Multiple Myeloma. Br J Haematol (2020) 189:904–7. doi: 10.1111/bjh.16443 PMC7275888 32026474

[16] Flores-Montero J Sanoja-Flores L Paiva B Puig N García-Sánchez O Böttcher S. Next Generation Flow for Highly Sensitive and Standardized Detection of Minimal Residual Disease in Multiple Myeloma. Leukemia (2017) 31:2094–103. doi: 10.1038/leu.2017.29 PMC5629369 28104919

[17] Maclachlan KH Came N Diamond B Roshal M Ho C Thoren K. Minimal Residual Disease in Multiple Myeloma: Defining the Role of Next Generation Sequencing and Flow Cytometry in Routine Diagnostic Use. Pathology (2021) 53:385–99. doi: 10.1016/j.pathol.2021.02.003 33674146

[18] Mazzotti C Buisson L Maheo S Perrot A Chretien M-L Leleu X. Myeloma MRD by Deep Sequencing From Circulating Tumor DNA Does Not Correlate With Results Obtained in the Bone Marrow. Blood Adv (2018) 2:2811. doi: 10.1182/bloodadvances.2018025197 30355580 PMC6234381

[19] Vij R Mazumder A Klinger M O’dea D Paasch J Martin T. Deep Sequencing Reveals Myeloma Cells in Peripheral Blood in Majority of Multiple Myeloma Patients. Clin Lymphoma Myeloma Leukemia (2014) 14:131–9.e131. doi: 10.1016/j.clml.2013.09.013 24629890

[20] Yukami H Nakamura Y Watanabe J Kotaka M Yamazaki K Hirata K. Minimal Residual Disease by Circulating Tumor DNA Analysis for Colorectal Cancer Patients Receiving Radical Surgery: An Initial Report From CIRCULATE-Japan. J Clin Oncol (2021) 39:3608–8. doi: 10.1200/JCO.2021.39.15_suppl.3608

