# Supplementary material for: Assessment of Molecular Residual Disease Using Circulating Tumor DNA to Identify Multiple Myeloma Patients at High Risk of Relapse
Source: Front Oncol. 2022 Feb 2;12:786451. doi: 10.3389/fonc.2022.786451 (PMC8848740; doi:10.3389/fonc.2022.786451)
Supplement: Supplementary file 1 [file DataSheet_1.docx]

Supplementary Information

*Brief Research Report*

**Assessment of molecular residual disease using circulating tumor DNA to identify multiple myeloma patients at high risk of relapse**

Binod Dhakal^1^, Shruti Sharma^2^, Mustafa Balcioglu^2^, Svetlana Shchegrova^2^, Meenakshi Malhotra^2^, Bernhard Zimmermann^2^, Paul R. Billings^2^, Alexandra Harrington^3^, Himanshu Sethi^2^, Alexey Aleshin^2^ and Parameswaran Hari^1^

1. Division of BMT & Cellular Therapy, Medical College of Wisconsin, Milwaukee, WI

2. Natera, Inc., Austin, TX

3. Department of Pathology, Medical College of Wisconsin, Milwaukee, WI

**Key words:** circulating tumor DNA **(**ctDNA), tumor-informed, minimal residual disease (MRD), multiple myeloma (MM), multiparameter flow cytometry (MFC)

**Short title:** ctDNA in multiple myeloma

**Corresponding author:**

Parameswaran Hari, MD, MRCP

Chief Professor of Medicine

Medical College of Wisconsin

Milwaukee, WI

Email: phari@mcw.edu

**Supplementary Figure 1.** MFC histograms showing residual disease from a patient. Residual myeloma cells (red) are CD38 (bright+), positive for CD20, CD117, CD200, and lambda light chain and negative for CD19 and CD56. Red: myeloma cells; magenta: hematogones; blue: B cells.
